# Electric-field facilitated rapid and efficient dissociation of tissues Into viable single cells

**DOI:** 10.1038/s41598-022-13068-6

**Published:** 2022-06-24

**Authors:** E. Celeste Welch, Harry Yu, Gilda Barabino, Nikos Tapinos, Anubhav Tripathi

**Affiliations:** 1 Center for Biomedical Engineering, School of Engineering, Brown University, Providence, RI 02912 USA; 2 Franklin W. Olin College of Engineering, Needham, MA 02492 USA; 3 Department of Neurosurgery, Warren Alpert Medical School, Brown University, Providence, RI 02912 USA

**Keywords:** Biomedical engineering, Electrical and electronic engineering, Tissue engineering, Diagnosis

## Abstract

Single-Cell Analysis is a growing field that endeavors to obtain genetic profiles of individual cells. Disruption of cell–cell junctions and digestion of extracellular matrix in tissues requires tissue-specific mechanical and chemical dissociation protocols. Here, a new approach for dissociating tissues into constituent cells is described. Placing a tissue biopsy core within a liquid-filled cavity and applying an electric field between two parallel plate electrodes facilitates rapid dissociation of complex tissues into single cells. Different solution compositions, electric field strengths, and oscillation frequencies are investigated experimentally and with COMSOL Multiphysics. The method is compared with standard chemical and mechanical approaches for tissue dissociation. Treatment of tissue samples at 100 V/cm 1 kHz facilitated dissociation of 95 ± 4% of biopsy tissue sections in as little as 5 min, threefold faster than conventional chemical–mechanical techniques. The approach affords good dissociation of tissues into single cells while preserving cell viability, morphology, and cell cycle progression, suggesting utility for sample preparation of tissue specimens for direct Single-Cell Analysis.

## Introduction

Single-Cell Analysis (SCA) is a growing field that endeavors to measure the properties of individual cells, while Single-Cell Sequencing (SCS) is a type of SCA that applies Next Generation Sequencing (NGS) technology to obtain genetic profiles of individual cells. These techniques are particularly applicable to heterogeneous cellular populations, such as cancer tissues, where rare mutations can drive metastasis and treatment response outcomes^1,2^. Despite the well-known phenomenon of tissue heterogeneity and the growing developments in SCS, clinical diagnostic workflows continue to rely on traditional approaches in which nucleic acids and other targets are extracted from bulk tissue and sequenced *en masse,* resulting in low resolution and poor detection of rare cell types^3^. The consequences of the loss of this information can be profound, including the possibility of patient misdiagnosis, with attendant morbidity and mortality^4^.

**Figure 1:**
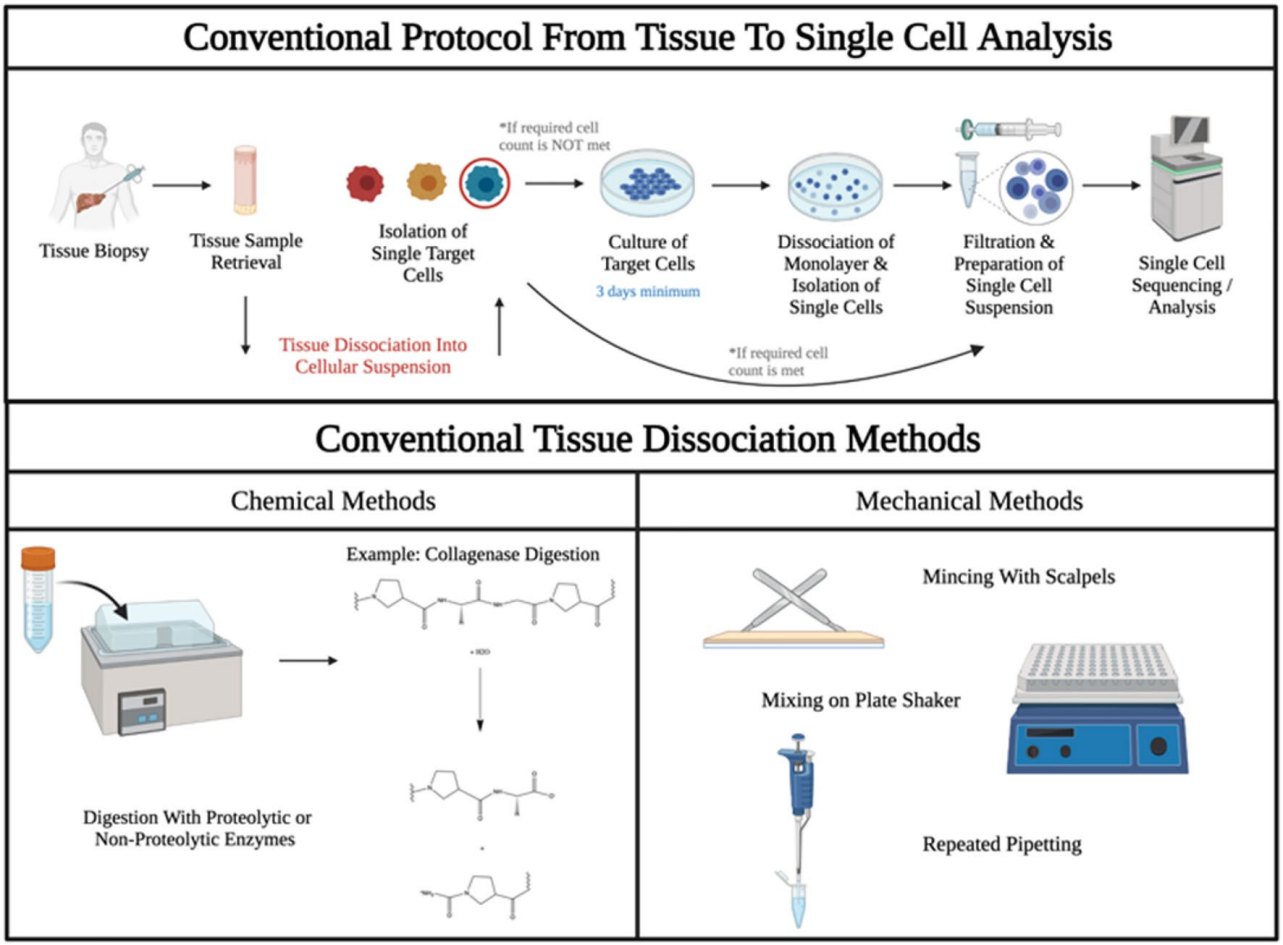
Overview of conventional protocol from tissue to single-cell analysis and tissue dissociation. The conventional tissue to single-cell analysis workflow is depicted, including the tissue dissociation into cellular suspension step, the focal point of this work. Post biopsy, tissue samples are recovered and subsequently dissociated, after which target cells are isolated. If baseline cell count values are not met, the cells are then cultured for a minimum of 3 days in standard protocols. In the case that the cells are cultured, the cellular monolayer is dissociated, and cells are again isolated. The cellular suspensions are then filtered of debris and doublet cells, yielding purified single-cell suspensions which can then be processed via single-cell sequencing. Traditional tissue dissociation methods highlighted here include chemical digestion with proteases, as well as mechanical mincing, mixing, and pipetting.

A major limitation of the clinical translation of SCS is the difficulty of isolating single cells from complex tissues. Current approaches require numerous time-consuming steps and can produce inaccurate genetic readouts due to the need to culture extracted cells prior to analysis if baseline cell counts are not met (Fig. 1).

All SCA technologies require efficient dissociation of tissue samples to afford a suspension of single cells. This frequently involves proteases and other reagents that disrupt cell–cell attachments. Often, external heating elements are used to produce a temperature of 37 °C to facilitate some enzymatic reactions, such as collagenase digestion. Mechanical actions like vortexing, shaking or centrifuging are used to enhance molecular dispersal and diffusion to the cellular boundaries and to provide forces that can be helpful in disrupting cell–cell attachments.

Conventional tissue dissociation can require hours of time and numerous instruments and reagents that must be stored and used under controlled conditions^5,6^. Attempts have been made to streamline, simplify and improve the disruption of complex tissues into their component cells. For example, Miltenyi Biotec has developed a tool (the GentleMACS Dissociator) that automates mechanical dissociation tasks. Nevertheless, these instruments can be low throughput and only partially effective, with incomplete dissociation of complex tissues and decline in cellular viability^7^.

Innovative microfluidic devices have also been created to improve the disruption of cellular aggregates into individual cells, often incorporating microfluidic flow against microscale objects such as pillars^8^, silica knives^9,10^ or mesh^11,12^. Alternatively, tailored mechanical shear forces and fluid jets can be applied within geometrically optimized microfluidic channels to disrupt cellular aggregates^13–15^. However, these devices are frequently prone to clogging, require pressure-driven flow and utilize complicated manufacturing processes.

Despite these innovations, the dissociation of tissues into single cells remains relatively unexplored, with limited technological progress at the commercial level. In this study we investigate the use of applied electric fields for the improved dissociation of tissues into their component single cells.

## Methods

### Electrical parallel plate electrode setup

The electrical setup consisted of the following components: a 2 mm gap length plastic encased electrode cell with two parallel plates, an adjustable electrical Direct Current (DC) power supply (Matsuda Precision) and voltmeter complete with two micro-electrodes, and a custom-made insulating holder. For oscillating voltage trials, a controlled wave function generator was used (BK Precision). The power supply was equipped with a controlled voltage function, which was used to maintain a uniform applied voltage to the parallel plate electrode cell over the duration of the various experiments. Regardless, voltage outputs were validated using a multimeter (Extech Instruments).

### COMSOL multiphysics modeling

In order to obtain a greater predictive understanding of the electric field within the parallel plate electrode cell, COMSOL Multiphysics modeling was performed using the AC/DC module. This software enabled modeling and simulation of the electric field and other electrical properties within the electrical setup. Full information is provided in the Supplementary Information.

### Media testing

When conducting the electrical experiments, a media test was first performed. In the first test, the gap in between the two metallic plates of the parallel plate electrode cell was filled with 300 µL of ultra-pure deionized water or DMEM media. Various phenomena, such as sample loss to bubbling, heating, conductivity, and pH fluctuation were then measured. This test was performed without any cells or tissue to assess liquid sample recovery in various electric field conditions for low conductivity (ultra-pure water) and high conductivity (DMEM media) solutions.

Preliminary tests examining the effect of suspension media on cells were then conducted using aliquots of MDA-MB-231 cells that were pre-counted using a hemocytometer. The same two solutions were tested, as well as a 300 mM sucrose solution that aimed to reduce the osmotic stress burden on cells in the ultra-pure water. The cells were exposed to the three conditions without any applied electric field and were examined at 5-, 15-, and 30-min timepoints using the live-dead staining protocol with flow cytometry. Viable cell recovery could then be assessed as a percentage, enabling a deeper understanding of when cell lysis and death begin to occur in various media. These experiments were both conducted prior to the electrical tissue dissociation experiments in order to optimize the media component of the workflow.

### Electrical dissociation protocol

Subsequent < 5-min tests were performed using 300 µL of LCMS grade ultra-pure filtered water, unless otherwise indicated. A 1 mm diameter tissue biopsy core was taken from a bovine liver tissue specimen as described previously using a Robbins Instruments biopsy tool^6^. The biopsy core was then loaded vertically into the cavity between the electrodes, and positioned equidistant from, but not touching the two electrodes.

**Figure 2:**
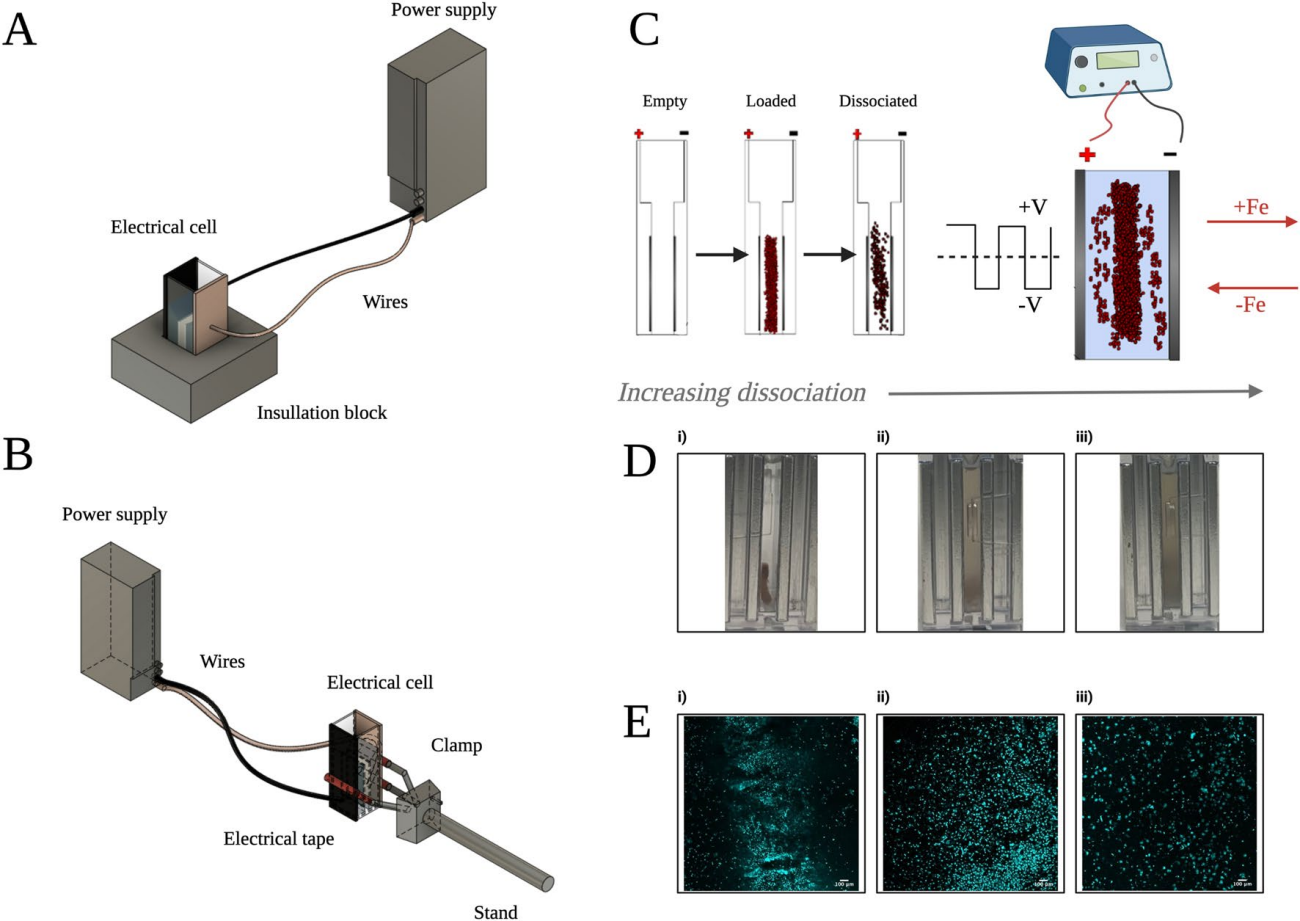
Schematic of the electrical tissue dissociation device configurations and dissociation process. (**A**,**B**) illustrate the different electrical device configurations used in the short time course (< 5 min) and long-time course (< 30 min) trials, respectively. (**C**) Schematic representation of the process of electrical dissociation. Pictures of dissociating tissue within 2 mm gap are represented in (**D**), while corresponding microscopy images taken in real time are represented in (**E**). (**Di**,**Ei**) represent a baseline of dissociation corresponding to having just submerged the tissue. A small number of surface cells are immediately washed off. (**Dii**,**Eii**) represent ~ 50% dissociation of the tissue, while figures (**Diii**,**Eiii**) represent ~ 100% dissociation of the tissue using applied electric fields. All qualitative results presented were quantitatively validated using flow cytometry.

In < 5-min experiments, the electrode cell was then positioned within the insulating holder, and the electrode wires were placed at either side of the device, putting them in contact with each respective metal plate (Fig. 2A). The power supply, which was pre-set to a specific voltage of interest, was then turned on. Actual voltages and amperages were independently verified using a multimeter. The voltage was controlled within the experiments. and different voltages were tested, as expressed in electric field strengths of 10, 20, 30, 40, 50, 60, 70, 80, 90 and 100 V/cm. Actual voltages of 2, 4, 6 V etc. were applied to the plates in order to achieve these conditions. These electric field strengths and voltages are well below the established electroporation threshold^16^.

At different time intervals over a period of 5 min, the voltage was automatically stopped and the entire 300 µL liquid sample of dissociated cells in suspension was withdrawn from the device using a sterile 20 Gauge needle. Trials were replicated at least ten times in order to verify experimental reproducibility.

For long time course < 30-min trials only, an alternative setup was developed (Fig. 2B). The setup used a holding rack in order to secure the electrodes in place over time. The electrodes were secured to the parallel plates on either side of the cell using the rack. Electrical tape was used to ensure insulation. Water was supplemented with 300 mM sucrose.

For oscillating voltage trials, the information was programmed into the function generating power supply system, and the experiment was left to run in the same manner as the 0 Hz DC voltage experiments. A square wave function was used, in which the peak of the wave was equal to the maximum voltage and the trough was equal to the minimum voltage of equal magnitude (Fig. 2C). Various frequencies were tested, including a lower limit of 10 Hz and upper limit of 1 MHz, based on the frequency limitations of the function generator.

Immediately after all treatments, cells were pelleted so that the supernatant could be removed. They were then transferred into a solution containing PBS, in order to prevent cell lysis from hyperosmotic swelling.

Information on electrical dissociation phenomena and the physical theory of dissociation is provided in the Supplementary Information (Supplementary Eqs. S1–S4).

### Horizontally oriented device fabrication

A prototype microfluidic chip was created for the purpose of optically interrogating the phenomenon of electrical dissociation in real time. The chip enabled viewing of the dissociation phenomenon under a microscope, which was challenging in the vertical orientation (Fig. 2D,E). The chip was used solely for the purpose of visualizing the dissociation process and collecting images.

The chip was fabricated as follows: The electrodes were removed from the vertical parallel plate electrical cell and sealed onto a custom optically transparent glass slide chip, which was itself placed into an imaging dish. Wires were then attached to the sides of the electrodes in order to transmit voltage from the power supply. The original electrodes and gap length were maintained in order to limit experimental variability. The device could be fitted with tubing at either end to retrieve sample more effectively via a simple pump, or the sample could be withdrawn as before, using a syringe.

### Tissue and cell sources

Bovine liver tissue was utilized in dissociation characterization tests using a previously reported protocol^6^. The tissue was obtained from a local butcher and promptly cryopreserved for later analysis.

As live cells were needed to examine effects on viability, MDA-MB-231 triple-negative breast cancer cells were cultured for use. The MDA-MB-231 cells were cultured in media consisting of Corning DMEM with L-glutamine, 4.5 g/L glucose, and sodium pyruvate supplemented with 10% fetal bovine serum and 1% Penicillin-Streptomycin^6^.

Partial passage was used to elute three-dimensional cellular clumps, which were tested in the dissociation workflow for the purpose of examining viability of live cells only. These cells were not used to assess *ex vivo* dissociation efficacy due to their limited complexity in comparison to *ex vivo* tissues.

In order to test the applicability of the process on different tissues, human clinical glioblastoma (GBM) tissues were obtained from patients at the Rhode Island Hospital under Brown University IRB #405,616. These tissues are notably more complex, and more difficult to dissociate when compared to the liver tissue^17,18^. The GBM tissues were analyzed immediately following their removal from patients in order to simultaneously assess dissociation and viability. The tissues were sectioned into pieces with the same dimensions as tested with the bovine liver tissue, and then exposed to various dissociation protocols.

### Flow cytometry

Flow cytometry was used in the bovine liver tissue experiments to assess the total number of dissociated cells across various electrical treatments and compare these results to control and chemical/mechanical treatments. Chemical treatments consisted of treating with 1% collagenase at 37 °C. Cell counting in flow cytometry was performed using a previously described size gating method^6^. This method of inferring cell size using size-controlled flow cytometry beads and cell type bins was combined with an additional layer of security. An extra step was added to the sample preparation protocol by treating the dissociated cells with red blood cell lysis buffer, DNAse I, and then staining with Hoechst 33342 to nonspecifically stain the nuclei of cells. This method enabled quantification of tissue cells while distinguishing them from cellular debris and other particles within the sample.

#### DNAse I treatment

DNAse I solution was prepared by combining 327 µL of nuclease free water with 60 µL of DNAse I buffer (PerkinElmer) and 3 µL of DNAse I stored in glycerol. After preparation, the stock solutions were stored in a 4 °C refrigerator.

The cellular suspension was spun down using a centrifuge at 1,500 RPM to form a cellular pellet. The supernatant (~ 300 µL ultra-pure water) was then removed with a pipette, while being careful not to disturb the pellet. The cells were treated with 20 µL of DNAse I. After the DNAse I solution was pipetted onto the cells, the cells were resuspended out of the cell pellet and into the solution via gentle agitation.

The solution was then incubated with the cells for 5 min, centrifuged, and removed with a pipette. After the DNAse solution was removed, the cells were then resuspended in ~ 248 µL of PBS and 2 µL DNAse solution, which served as a recommended low maintenance concentration. The tube was gently agitated to evenly disperse the cells.

#### Hoechst 33342 staining for flow cytometry

The Hoechst 33342 stain is a readymade product for flow cytometry live/dead staining purchased from ThermoFisher Scientific (Hoechst 33342 Ready Flow Reagent, Thermofisher Scientific). Instead of dropping the dye, a more quantitative approach was taken by pipetting the dye in known volumes and concentrations in order to reduce variability. A concentration of 1 µg/mL was utilized, as recommended by ThermoFisher Scientific.

The DNAse treated cell solution was split into 2 aliquots of 125 µL used in flow cytometry analyses in replicates. The stain was placed at the recommended concentration within each sample tube. The tubes were then incubated at 37 °C for 30 min with the stain. Afterwards, the contents of the tubes were pipetted onto 96 well plates. The plates were filled with an equal volume of PBS to a volume of ~ 250 µL, 125 µL of which was analyzed on the flow cytometer.

#### Mathematical modeling of expected cell numbers

Dissociation efficacy was quantified using a previously reported comprehensive methodology^6^ that employs a combination of techniques in order to examine the efficacy of dissociation and cellular retrieval^19,20^. Cell count estimates based on surface area and weight of bovine liver tissue specimens are synthesized into a single mathematical model which was used to calculate the percent dissociation for each sample (Supplementary Eq. S5). Prior to this work, the model was established to have a Pearson R-squared correlation value of 0.93 and 2-tailed *p* value < 0.001 when comparing the theoretical predicted values to the experimentally obtained values (Supplementary Fig. S1).

### Microscopy viability assay

Viability tests were performed on live, freshly passaged MDA-MB-231 triple negative breast cancer cells. The cells were exposed to the same electric field conditions as tissue sections. They were microscopically examined using both hemocytometry and fluorescence microscopy in order to assess cellular integrity and viability. Live and dead cells were quantified using the Image Processing Workflow described below and characterized in part elsewhere^6^.

An Olympus FV3000 confocal microscope (Brown University Leduc Bioimaging Facility) was used to assess viability and membrane integrity of MDA-MB-231 cells. A chemical and mechanical control using 1% collagenase at 37 °C was compared to both a 100 V/cm DC electric field condition, as well as a 1 kHz oscillating voltage condition with the same electric field strength.

Hoechst 33342 was again used as a nonspecific stain, while DRAQ7 was used as a “dead” stain. An anthracycline derivative, DRAQ7 enters through cells with compromised membrane integrity, binding to DNA^21^. It can be useful in the real-time monitoring of cell death, as it does not induce death, but serves as an effective marker of compromised membrane integrity^21^. These two dyes were co-stained for fluorescent microscopy “live/dead” analyses, and 10 µL samples were placed onto imaging dishes for investigation at 10X, 20X, and 100X oil-immersion.

### Mitotic cell cycle assay

An assay for mitotic cells and cell cycle progression was conducted using an established protocol^22^. As cell cycle disruption at the mitotic exit phase has been observed with higher frequency electric field treatments (e.g. 200 kHz), it was important to examine whether this effect occurs here^22–25^.

Cellular suspensions of MDA-MB-231 were either not treated or electrically treated at 100 V/cm 1 kHz. Afterwards, Anti-phospho Histone H3 (Ser10) Antibody AlexaFluor488 Conjugate (Sigma-Aldrich) was used to stain selectively for phosphorylated Histone H3, an indicator of mitosis. A recommended 1:50 dilution of antibody was prepared in PBS, and co-incubated with cells at 37 °C for 60 min. 10 µL samples of cells were then visualized under the confocal fluorescence microscope in imaging dishes and images taken for analysis at 10X. Cell-count matched samples were also analyzed for relative fluorescence intensity using a ThermoFisher Nanodrop 3300 fluorospectrometer.

### Image analysis platform

The ImageJ—FIJI image analysis software was used for the purposes of cell counting from confocal microscopy images, morphology assessment, and live dead image processing (National Institutes of Health). A workflow utilized in our previous work was again used for visual processing^6^.

This same image analysis workflow was used both for image processing of cells stained with a single fluorescent probe, as well as images with two different fluorescent probes. Images with two different probes required a simple additional step of discerning between different fluorophores by setting fluorescence thresholds before proceeding with the cell counting workflow.

### Human clinical glioblastoma tissue testing

The protocol developed and optimized using bovine liver tissue and MDA-MB-231 cells was then tested in order to assess its applicability across different tissue types. Freshly collected human clinical glioblastoma tissues were used as a model, due to their high complexity and difficulty of dissociation.

Tissue sections with the same dimensions (~ 1 mm × 5 mm) were collected. Sections with necrosis were removed from analysis to limit confounding results. Sections were then exposed to the dissociation conditions. Control untreated sections were placed in Complete media consisting of 1X Neurobasal A, 2 mM GlutaMAX-I supplement, 100X Anti-Anti, 20 ng/mL bFGF and EGF, B-27A and Heparin. Control dissociation treated sections were exposed to the routine protocol with which these sections are generally dissociated, consisting of manual mincing followed by chemical treatment with 1% collagenase and dispase solution while manually agitating the tube. This protocol took approximately 60 min to complete. Electrically treated sections were exposed to the 100 V/cm 1 kHz electrical condition for a total of 15 min.

After treatment, the suspensions were immediately spun down and red blood cell lysis was performed using 1X Red Blood Cell Lysis Buffer (ThermoFisher) following established ThermoFisher Scientific best practices. After the lysis step, the suspensions were spun down again and resuspended in the Complete Media solution. GBM tissue dissociation was assessed using a Countess Automated Hemocytometer following established protocols in order to simultaneously gather information on cellular recovery from tissues and viability of recovered cells.

### cfDNA assay

The cfDNA in dissociated bovine liver tissue samples untreated with DNase I was analyzed at 5-, 15-, and 30-min time points in an untreated control condition and various electric field oscillation frequencies to assess whether genetic contents are released from cells during the electrical treatment. All cells were removed from the 300 µL solution, leaving only the supernatant. The QIAGEN QIAamp Circulating Nucleic Acid Kit was then utilized to extract and prepare the circulating nucleic acids, and RNase digestion was performed to purify the cfDNA. The cfDNA was subsequently quantified by dropping 1 µL onto the Nanodrop 1000 Spectrophotometer and measuring absorbance at 260 nm with respect to 280 and 230 nm.

### RNA analysis

Samples of 500,000 MDA-MB-231 cells each were exposed to either a control consisting of no treatment, an optimized chemical/mechanical treatment, or an optimized electrical treatment of 100 V/cm 1 kHz. Other samples were exposed to the optimized electrical treatment and then added to media and placed in a thermal controlled CO_2_ incubator for 15 and 60 min, respectively, to assess the effect of a “recovery period”.

After treatment, the cells were pelleted via centrifugation and RNA was extracted from the populations using the QIAGEN RNeasy Micro Kit. DNAse I digestion and RNA cleanup were performed using the same spin column kit, following QIAGEN protocols. The RNA was eluted into 30 µL of nuclease free, RNase free water. The column was then washed with an additional 30 µL of water.

Total RNA was then quantified using the Nanodrop 1000 Spectrophotometer by dropping 1 µL of the RNA sample onto the pedestal and again measuring absorbance at 260 nm with respect to 280 and 230 nm. The Agilent 2100 BioAnalyzer was then utilized for confirming total RNA concentration and ascertaining the RNA Integrity Number (RIN) with the RNA Nano chip.

Samples were adjusted to the same RNA concentration by adding RNase free water or utilizing controlled evaporation centrifugation. Reverse transcription of RNA into cDNA was then performed using the Applied Biosystems High-Capacity cDNA Reverse Transcription Kit. qPCR was performed for 6 probes which have an established relationship to MDA-MB-231 stress-response—SERPINE1, INHBE, FLRT1, HSPA5, ECM2 and PLAT^26^. Custom ThermoFisher TaqMan probes were created to amplify these targets in qPCR. Expression changes were examined in the chemical/mechanical and electrical treatment groups in comparison to the untreated control-established baseline.

### Statistical analysis

All studies were performed with a minimum of 5 biological and 3 technical replicates. Results are represented as average ± standard deviation. Where relevant, a one-way analysis of variance (ANOVA) or a two-way ANOVA was performed. Tukey’s post-hoc test and 95% confidence interval were used for analysis. Multiple comparison analysis was used to assess relationships between variables. *p < 0.05, **p < 0.01, ***p < 0.001, ****p < 0.0001. All statistical tests were conducted using GraphPad Prism software.

## Results

### Physical modeling of electric fields

**Figure 3:**
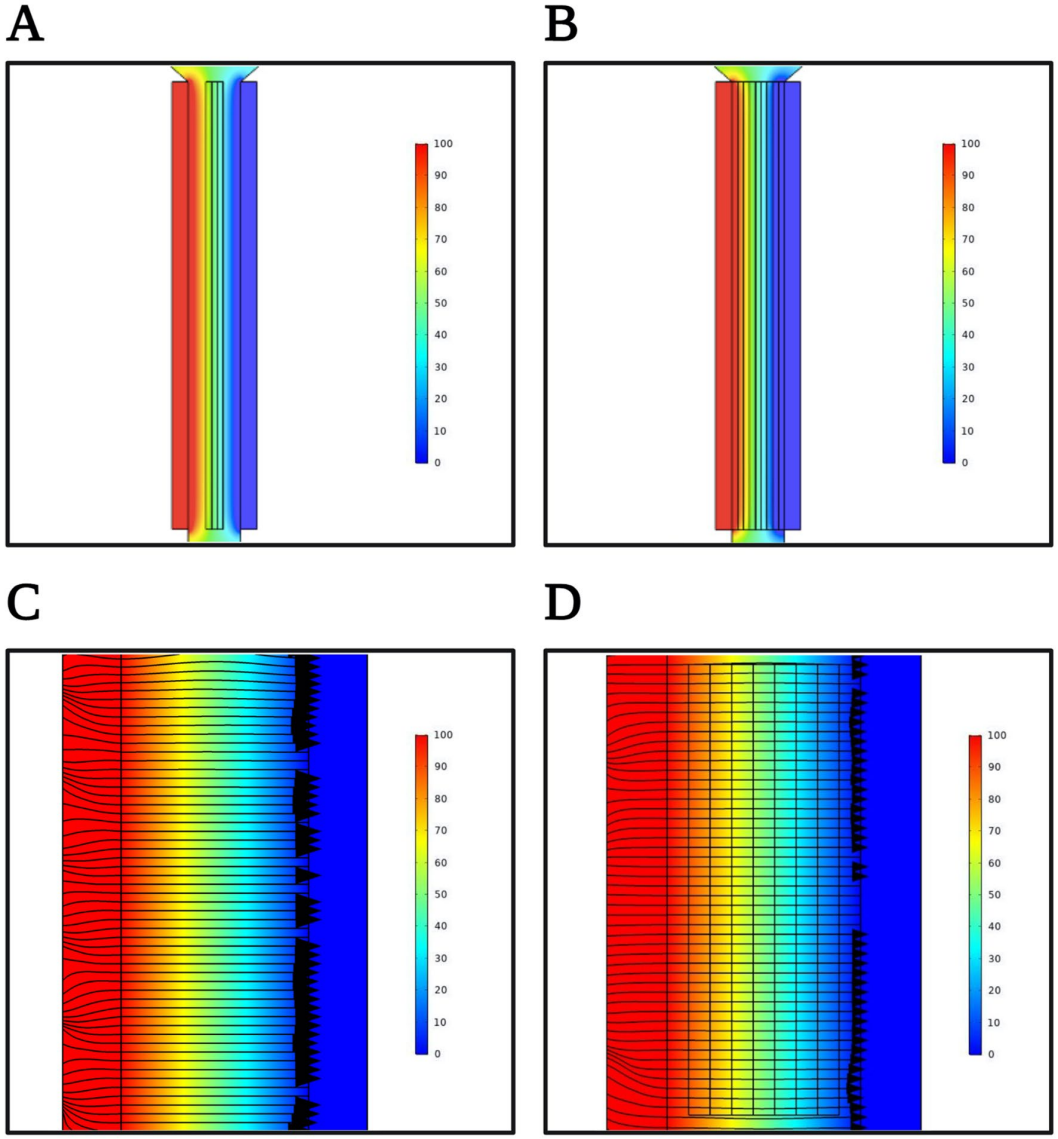
COMSOL finite element modeling of electrical setup. (**A**) Illustrates the electrical model with 3 simulated tissue layers and (**B**) illustrates the electrical model with 9 simulated tissue layers. The tissue layers decrease in conductivity and increase in permittivity to simulate actively dissociating tissue cores. (**C**) Uniform electric field lines in electrical cell without simulated tissue, illustrating the linearity of the electric field across the cavity. (**D**) Uniform electric field lines in electrical cell with simulated tissue model, illustrating that the electric field linearity is not disrupted by the presence of tissue within the cavity.

In this work, we developed a method of dissociating tissue into cells using applied electric fields. Prior to experimentally investigating parameters of electrical dissociation, we created a physical model to assess whether electric-field linearity would be preserved within the parallel plate electrode cell (Supplementary Information). Tested voltages of 2–20 V were applied across the electrodes and through a simulated cavity with just water as well as water with a simulated tissue core, and water with 3–9 layers of simulated dissociating tissue (Fig. 3A,B). The electric field was linear between the electrodes across all tested conditions, was not deflected by the tissue, and did not create any hot spots within the cavity (Fig. 3C,D).

### Effect of media composition on sample recovery

The effect of media composition on sample recovery and sample loss due to bubbling was investigated prior to conducting comprehensive tissue dissociation studies. Ultra-pure water was compared to media in order to assess relative recovery of respective liquid samples (Supplementary Fig. S2). Water was used in this context as a low-osmotic strength and low-conductivity solution, while the media, which contained several added salts and ions, represented a higher osmotic strength and conductivity solution. The Debye length of the water was approximately 10X that of the media. The media also had a notably higher viscosity. Other buffers such as 300 mM sucrose are examples of how solutions can be made isotonic to cells while retaining their low conductivity.

Although much less significant in water trials, low-level bubbling was also observed to take place in higher DC electric field strengths such as 100 V/cm, resulting in 36 ± 11% sample losses at 5 min in comparison to 78 ± 10% (Supplementary Fig. S2A). Other research has shown that, below 1–10 kHz, electrolysis is frequently observed in low conductivity solutions^27^. This may help to explain the excessive bubbling which led to low sample recovery in the 100 V/cm treatment with no oscillation, and why 1 kHz oscillation frequency showed improved results of only 8 ± 7% losses after 5 min (Supplementary Fig. S2B). While bubbling does result in mechanical agitation via induced turbulence, too much bubbling results in sample loss, and electrolysis has been shown to potentially decrease cellular viability^27^.

It was found that high salt/ion content in the media resulted in increased conductivity, elevated temperature (presumably due to Joule heating) and pronounced bubbling, causing low sample recovery and cell death in viability flow cytometry and microscopy studies with MDA-MB-231 cells (Supplementary Fig. S3). In contrast, pure water solutions exhibited none of these deleterious effects. However, placing cells into hypotonic environments for prolonged periods is known to lead to bursting of cells by osmotic pressure. Despite this, experiments showed that brief (< 5 min) treatment times in ultra-pure water did not significantly reduce cell viability (Supplementary Fig. S4). Furthermore, long time courses of more than 15 min could be supplemented with 300 mM sucrose to maintain osmolarity and prevent cellular bursting and viability loss (Supplementary Fig. S4). Ultimately, water and sucrose supplemented water were seen to be more effective candidates for the dissociation of tissues, liquid sample recovery, and preservation of cellular viability, so long as the cellular samples were rapidly removed and immersed in an isotonic PBS solution for further analysis.

### Experimental tissue dissociation results

#### Effect of DC electric fields on tissue dissociation

**Figure 4:**
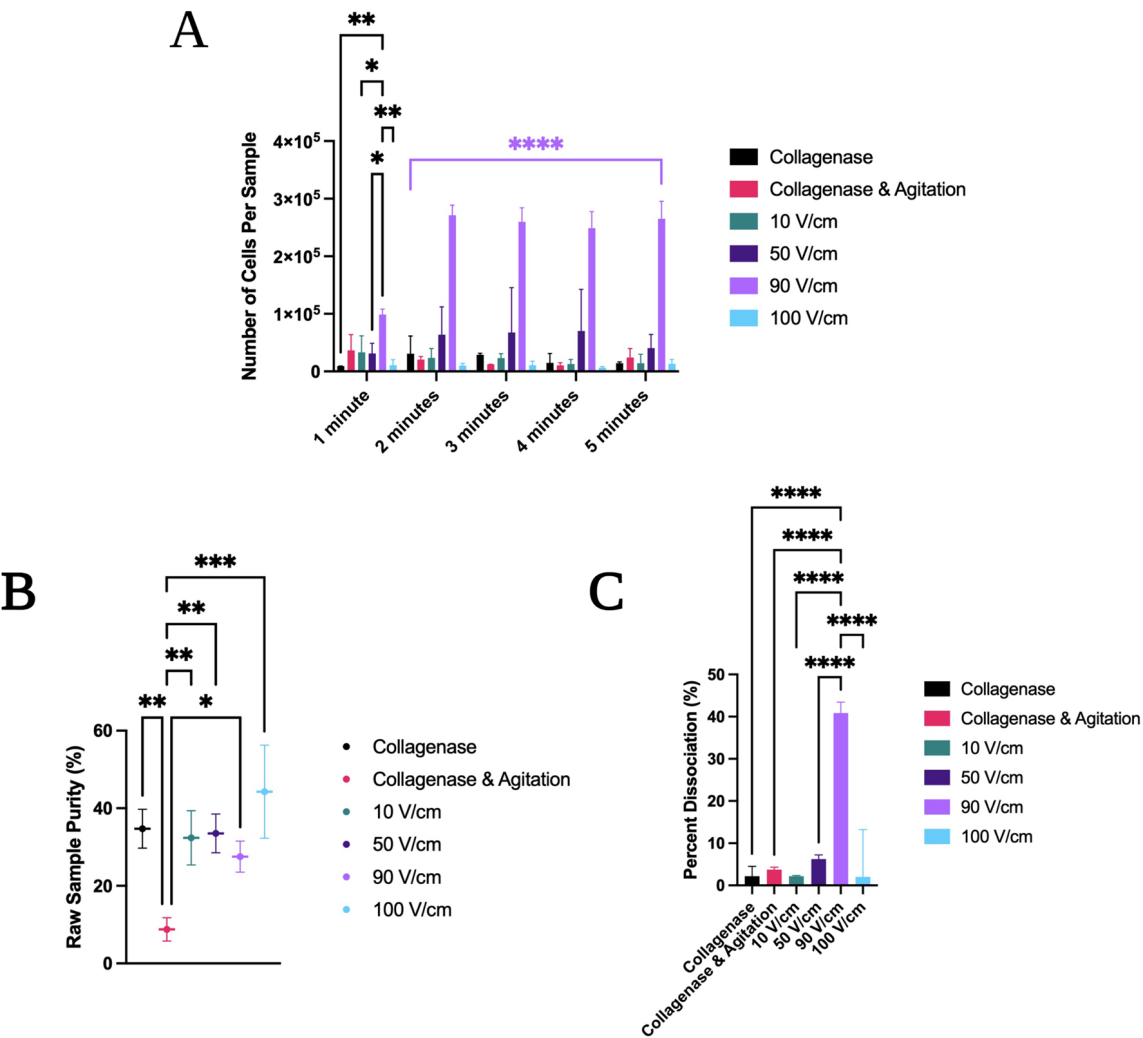
Examination of dissociation efficacy at electric field strengths of 10–100 V/cm over a short (< 5-min) time course. (**A**) Raw number of cells processed in a given sample via flow cytometry across various DC electric field conditions as well as control collagenase and collagenase with mechanical agitation conditions in a 5-min time course. 90 V/cm trials were significantly more effective at dissociating tissue across 2–5-min timepoints (*p* < 0.001). **B**) Sample purity values at 5 min for the same electrical, chemical, and mechanical conditions. (**C**) Percent dissociation of tissues at 5 min. 90 V/cm trials were significantly more effective at dissociating tissues (*p* < 0.001). All quantitative results were collected using flow cytometry. One-way ANOVA with Tukey post-hoc analysis and a 95% confidence interval was performed for samples (**A**) across a time course and (**B**,**C**) at the 5-min time point. N ≥ 10, *p < 0.05, **p < 0.01, ***p < 0.001, ****p < 0.0001. Information on nonsignificant results is not illustrated on the graphs. Colored bars and asterisks represent significance trends across numerous timepoints for a given electrical condition when compared to all other electrical conditions. All significances between 100 V/cm 1 kHz and all other treatments are ****p < 0.001 from 2 to 5 min.

The efficacy of electrical dissociation of tissue into single cells was first assessed at lower-level DC electric field strengths between 10 and 100 V/cm over a 5-min time course using bovine liver tissue in the first electrical setup (Fig. 2A). Various metrics were applied to determine a comprehensive understanding of electrical dissociation across different conditions. First, the raw numbers of single and aggregated target tissue cells were retrieved, as measured via flow cytometry (Fig. 4A)^6^. Sample purity was assessed by looking at the number of tissue cells with respect to all other particles in suspension, including things like extracellular matrix fragments (Fig. 4B). Finally, this data was used to determine the percent dissociation using the bovine liver tissue compositional model (Fig. 4C)^6^. Cellular debris in the sample was excluded from the analysis.

In the initial short (< 5-min) time course, maximum percentage dissociation was obtained at 41 ± 3% for applied electric field strengths of 90 V/cm. While there was some variability across time, similar recovery was obtained even after a short duration of 2 min in higher applied field strength trials (e.g. 90 V/cm). It is also notable that dissociation was most effective in the 100 V/cm samples, but samples had a tendency to bubble over at E-field strengths of 100 V/cm with non-oscillating voltage in the first experimental setup, which significantly reduced cellular recovery.

Despite reduced cellular recovery due to bubbling in the 100 V/cm condition, this electric field strength afforded the highest sample purity of 44 ± 12%. Sample purity represents a metric of the ratio of single cells to total particles, including cellular aggregates, fragments, debris, and other constituents. This was notably significantly higher than that of the collagenase and mechanical agitation treatment, which surprisingly had the lowest purity of all tested samples at 9 ± 3%, likely due to the presence of ECM fragments or cellular lysis resulting from chemical or mechanical damage.

In order to determine if increase in treatment time can afford an increase in cellular recovery from tissue cores, dissociation efficacy was examined again at field strengths from 10 to 100 V/cm, using a constant non-oscillating voltage, but over a longer (< 30 min) time course at intervals of 5, 15, and 30 min. Representative E-field strengths of 10, 50 and 100 V/cm were studied in this manner. Additionally, the second electrical setup was used for long time course trials to facilitate hands-off processing (Fig. 2B).

**Figure 5:**
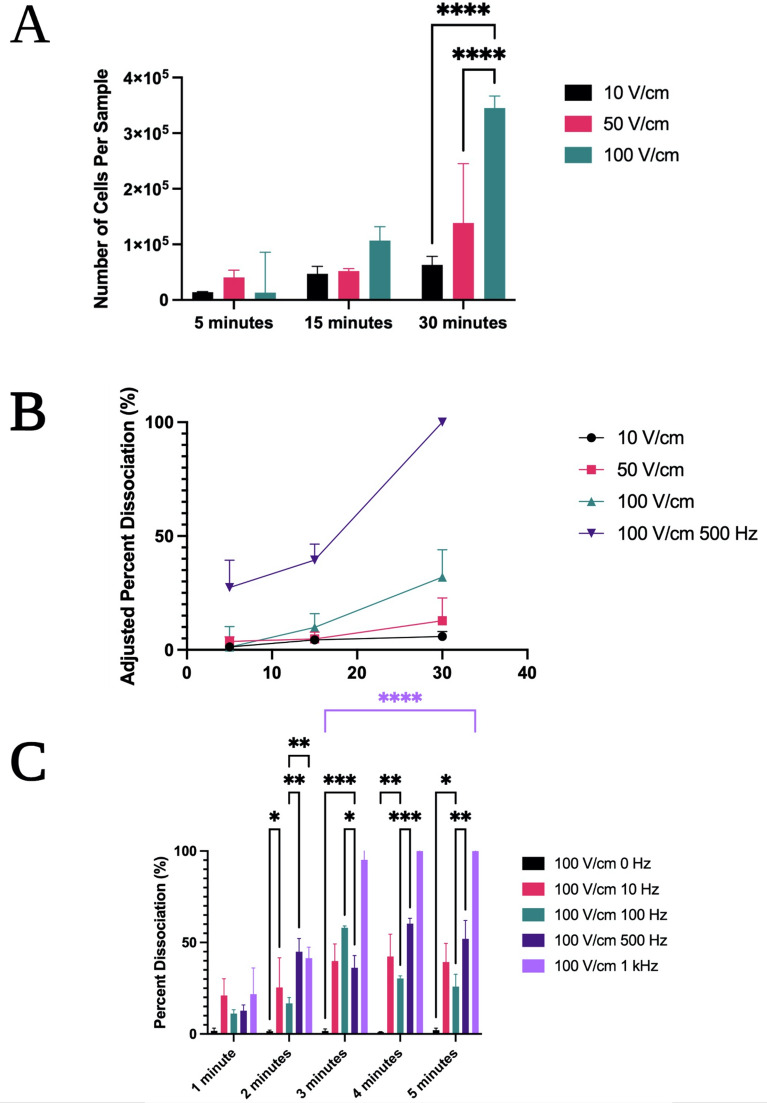
Examination of dissociation efficacy at electric field strengths of 10–100 V/cm over a long (< 30 Min) time course and with various frequencies of oscillation. (**A**) Raw number of tissue cells isolated in subsamples with constant DC electric fields at a long time course of < 30 min. (**B**) Represents percent dissociation with and without oscillating voltage over a long time course of < 30 min. (**C**) Represents a comparison of percent dissociation in oscillating voltage trials over a short time course of (< 5 min). Two-way ANOVA with Tukey post-hoc analysis and a 95% confidence interval was performed. N ≥ 10, *p < 0.05, **p < 0.01, ***p < 0.001, ****p < 0.0001. Colored bars and asterisks represent significance trends across numerous timepoints for a given electrical condition when compared to all other electrical conditions. Unless otherwise denoted, significance between 100 V/cm 0 Hz treatments and all other treatments is ****p < 0.0001 from 2 to 5 min. All significances between 100 V/cm 1 kHz and all other treatments are ****p < 0.001 from 2 to 5 min.

The dissociation results at these constant non-oscillating voltages were quite mild, even over a longer period of 30 min – 32 ± 12% dissociation was observed after 30 min in the 100 V/cm DC treatment, with reduced cellular recovery due to bubble formation again observed in both the 50 V/cm and 100 V/cm trials (Fig. 5A). This suggests that longer treatments with constant E-fields is not an efficient processing strategy to improve tissue dissociation compared to treatments of shorter length.

#### Effect of square-wave oscillation on tissue dissociation

Subsequently, oscillating voltage was tested as a method to reduce bubble formation and improve processing speed and recovery. The use of oscillating square wave voltages with varying frequencies was found to not only reduce the formation of bubbles, but to significantly improve tissue dissociation across all time points from 2 min onward. In a long-term time course, significantly more cells were recovered at 5-, 15-, and 30-min timepoints when applying a 100 V/cm electric field at an intermediate 500 Hz frequency of oscillation while using a square wave function generator, roughly 91 ± 9% in comparison to 32 ± 12% (Fig. 5B). Highly effective dissociation of the tissue was therefore observed after 30 min had elapsed, with excellent cellular recovery. These results suggest that oscillating voltage may be a promising candidate for further investigation.

In order to more thoroughly assess the effect of oscillating voltage on cellular dissociation and optimize the process with regards to time, more trials were then conducted on shorter 1–5-min time points across a range of frequencies of oscillation (Fig. 5C). Within these trials, the applied electric field was held constant at 100 V/cm, while the frequency of oscillation was changed. Only square wave functions were used.

500 Hz showed similar results to the 15-min trial after as little as 2 min when switching back to the first device configuration (Fig. 5B,C). Notably, lower frequencies tended to produce less optimal results, consistent with the 100 V/cm results seen in non-oscillating voltage trials (Fig. 2D). 1 kHz frequency produced excellent dissociation in a rapid timeframe of < 5 min, with 95 ± 4% dissociation observed at 3 min.

In the 100 V/cm 1 kHz condition, the bovine liver tissue section dissociated completely into a cellular suspension within 4 min, which was immediately apparent by visual inspection. Flow cytometry results of dissociated cellular suspensions from the 1 kHz treatment showed excellent cellular recovery and no significant observed cellular fragmentation when examining the size gated results.

While the 30-min 500 Hz treatment on the second device and 5-min 1 kHz trial on the first device had similar dissociation efficacy, the lower time requirement of the 1 kHz trial is better suited to clinical translation of the electric field dissociation method. Furthermore, these results were comparable to a 15-min 1% collagenase/hyaluronidase and optimized mechanical plate shaking trial that was previously characterized as a best chemical/mechanical hybrid condition^6^.

#### Effect of tested electric fields on cell viability and morphology

**Figure 6:**
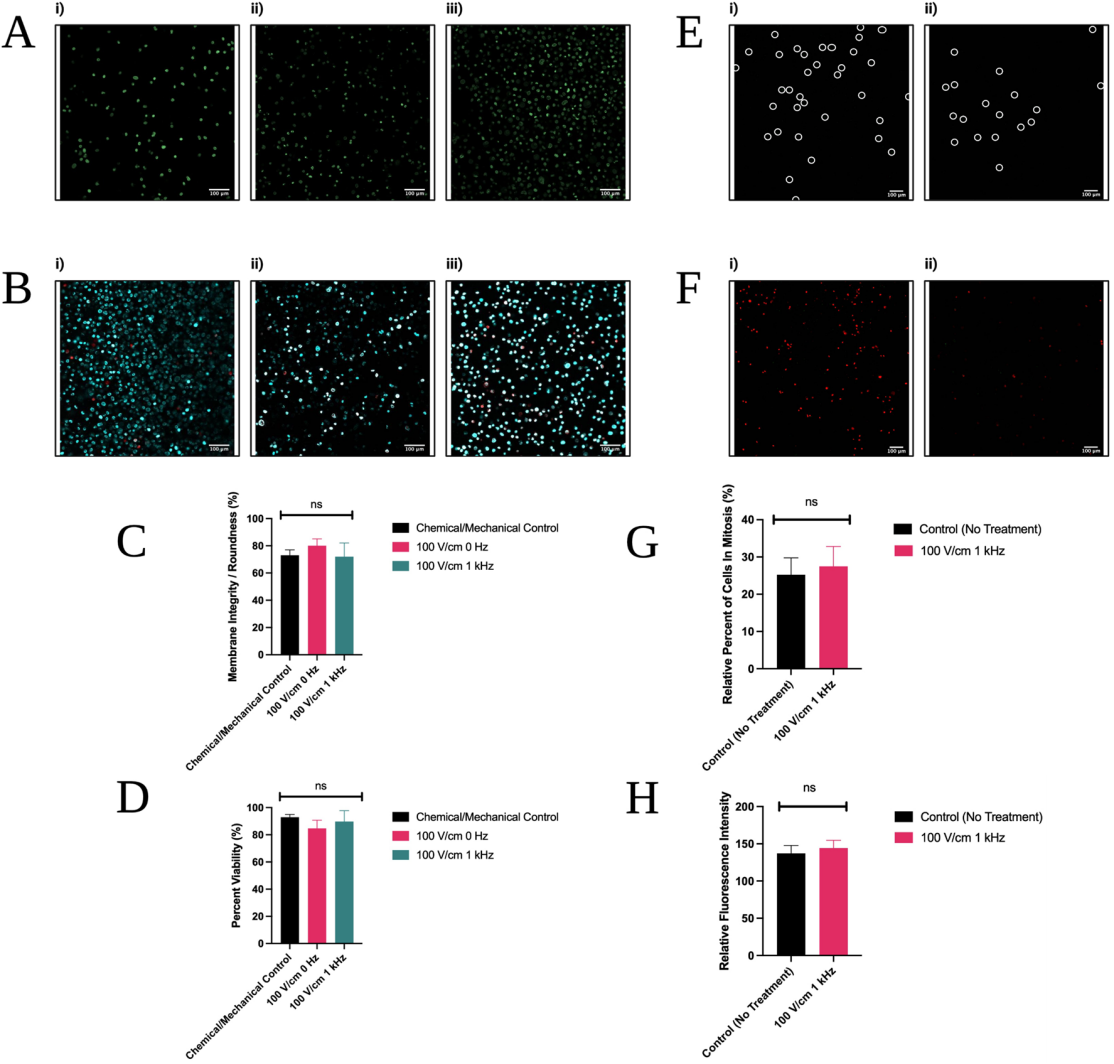
Human cancer cell results—quantity, viability, morphology, mitotic disruption. (**A**) Morphology images taken with a membrane permeable cell stain, Hoechst 33342. (**B**) Live/dead images taken with Hoechst 33342 as well as a membrane-impermeable dead cell stain, DRAQ7. (**Ai**,**Bi**) Represent samples subjected to the control chemical and mechanical treatment for 5 min. (**Aii**,**Bii**) Represent samples subjected to 100 V/cm 0 Hz treatment for 5 min. (**Aiii**,**Biii**) Represent samples subjected to 100 V/cm 1 kHz treatment for 5 min. (**C**) Represents extracted data analysis from images, comparing the best electrical condition to the control chemical and mechanical condition in order to assess morphology (*p* = 0.0755 for the control vs. 0 Hz condition, *p* = 0.9432 for the control vs. 1 kHz condition). (**D**) Uses the same approach to assess viability (*p* = 0.1547 for the control vs. 0 Hz condition, *p* = 0.4520 for the control vs. 1 kHz condition). (**E**) Cells stained with Anti-Phospho Histone H3 Ser10 Antibody AlexaFluor488 conjugate. (**F**) The same interrogation region but with fluorescent signal from cells stained with both the Anti-Phospho Histone H3 Ser10 Antibody AlexaFluor488 conjugate and non-specific dye to assess total number of cells. (**Ei**,**Fi**) are representative images for untreated control cells, while (**Eii**,**Fii**) are representative images of cells treated with the best electrical condition—100 V/cm 1 kHz. The results were quantitatively analyzed (**G**) with image analysis (*p* = 0.5464) and (**H**) with spectrofluorometry (*p* = 0.4701). All images were analyzed using ImageJ software. Electrical results were not significantly different when compared to chemical and mechanical controls according to One-way ANOVA with Multiple Comparisons testing for (**C**,**D**) and Welch’s t-test for (**G**,**H**).

In order to assess whether the electrical treatments significantly affect viability and morphology of cells, live MDA-MB-231 cells were exposed to the same electric fields as the biopsy specimens, and subsequently observed via confocal fluorescence microscopy. There was no statistically significant change in morphology (Fig. 6A,C) or decline in viability (Fig. 6B,D) observed, an indication that the low-level electric field treatment did not have a damaging effect on cells. All viability values were ≥ 85%—the chemical/mechanical control treatment produced 93 ± 2% viability, 100 V/cm 0 Hz treatment produced 85 ± 6%, and 100 V/cm 1 kHz produced 90 ± 8%. Their morphological roundness percentages were 73 ± 4%, 80 ± 5%, and 72 ± 10%, respectively—in line with what would be expected for this cell type. However, significantly more single cells were observed to have been recovered from electrical treatments after 5 min compared to chemical and mechanical treatments. In chemical–mechanical treatments, less cells were recovered and there were more remaining aggregates.

#### Effect of electric fields on mitosis and cell cycle progression

In order to assess whether a 100 V/cm 1 kHz electrical treatment disturbs cell cycle progression at mitosis, a conventional phosphorylated Histone H3 assay was conducted using an AlexaFluor488 conjugated antibody indicative of cells in mitosis. The assay was tested using live, fully passaged MDA-MB-231 cellular suspensions divided into two groups—untreated control, and 100 V/cm 1 kHz treated cells, both with 5-min trials.

The cells were then observed via confocal fluorescence microscopy. Images for Phospho-Histone H3 Ser10 cells were collected (Fig. 6E) and overlayed with images of all cells in all other phases with a nonspecific stain (Fig. 6F). After ImageJ processing and statistical analysis with Welch’s T-test, it was found that there was no statistically significant change in percent of cells in mitosis or observable effect on progression through the cell cycle (Fig. 6G). While untreated controls had 25 ± 4% of cells in mitosis, treated controls had 27 ± 5%. The test was found to be insignificant with a *p*-value of 0.5464. Spectrofluorometer RFIs were consistent with this trend (Fig. 6H).

#### Investigating electric-field based dissociation in clinical GBM

After initial characterization experiments were conducted to assess feasibility of electric-field dissociation in bovine liver tissue and MDA-MB-231, human clinical GBM tissues were tested in order to investigate the electrical technique’s feasibility in different tissue types and assess a more realistic application of the technology. Control preparations using manual minicing and chemical treatment with collagenase and dispase solution while manually agitating took an average of around 60 min, while electrical dissociation time courses were 15 min only.

**Figure 7:**
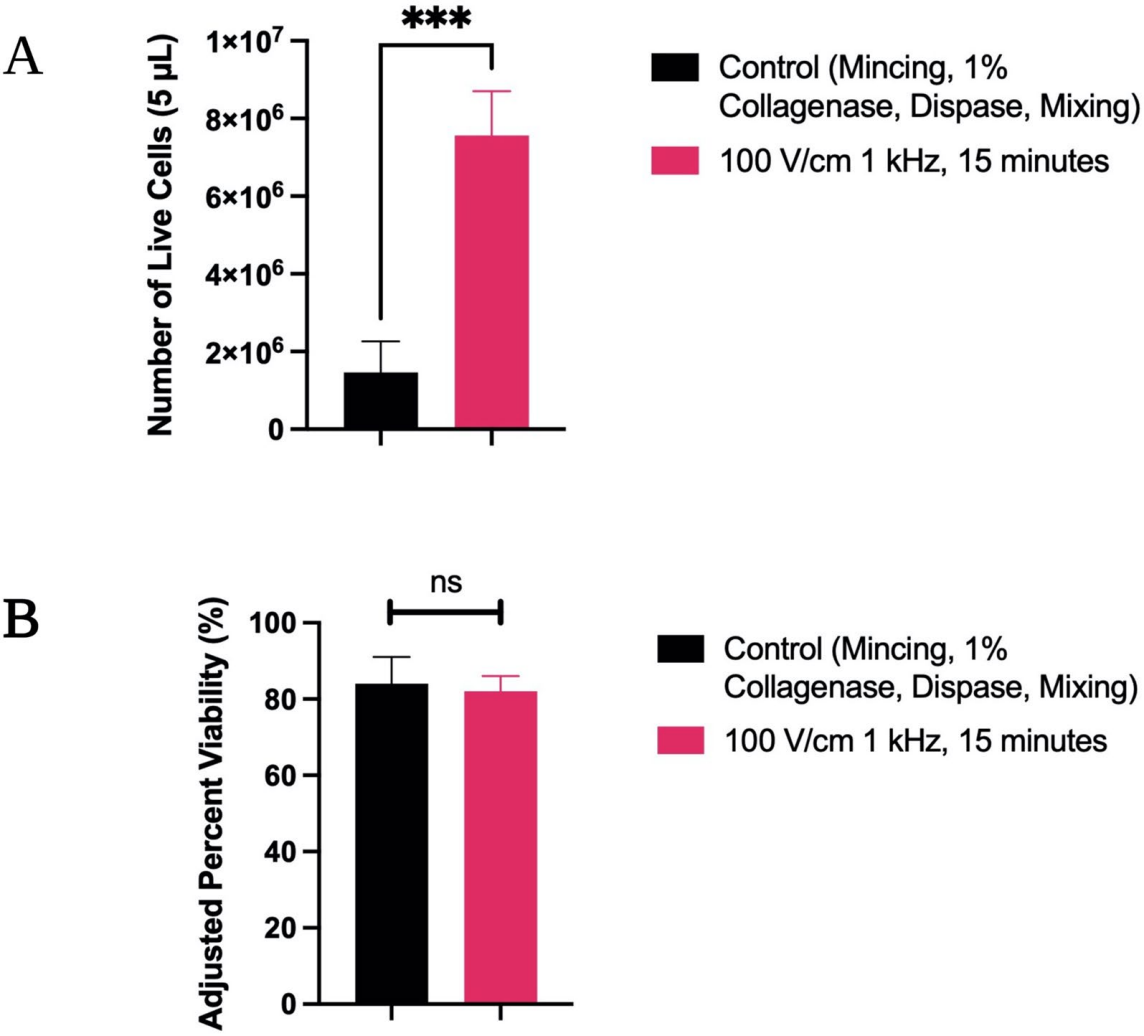
Human clinical glioblastoma results. (**A**) Illustrates the number of live cells isolated from size and volume-matched tissue sections dissociated using control and electrical protocols. The electrically treated group had a statistically significantly higher number of live cells than the control preparation with *p* < 0.001. (**B**) Illustrates the percent viability of chemical/mechanical and electrical preparations, as adjusted using an untreated control. The treated groups were not found to be significantly different in terms of the viability. All data on cellular recovery and viability was collected using a Countess Automated Hemocytometer.

Dissociation efficacy was assessed by looking at the number of live cells recovered in samples of liquid dissociate when standardizing tissue sizes and liquid volumes (Fig. 7A). Electrical treatments recovered slightly greater than 5 times as many live cells as traditional treatments in 1/4th of the time. Cellular recovery was statistically significantly higher across all electrical treatments, with *p* < 0.001.

When looking at viability as adjusted to an untreated control, the electrical and chemical/mechanical control treatment were not significantly different from one another (Fig. 7B). This is consistent with the previously discussed findings, in which higher cellular recovery is obtained with the electrical dissociation protocol, while viability is not significantly altered.

#### Effect of electric fields on cfDNA release

**Figure 8:**
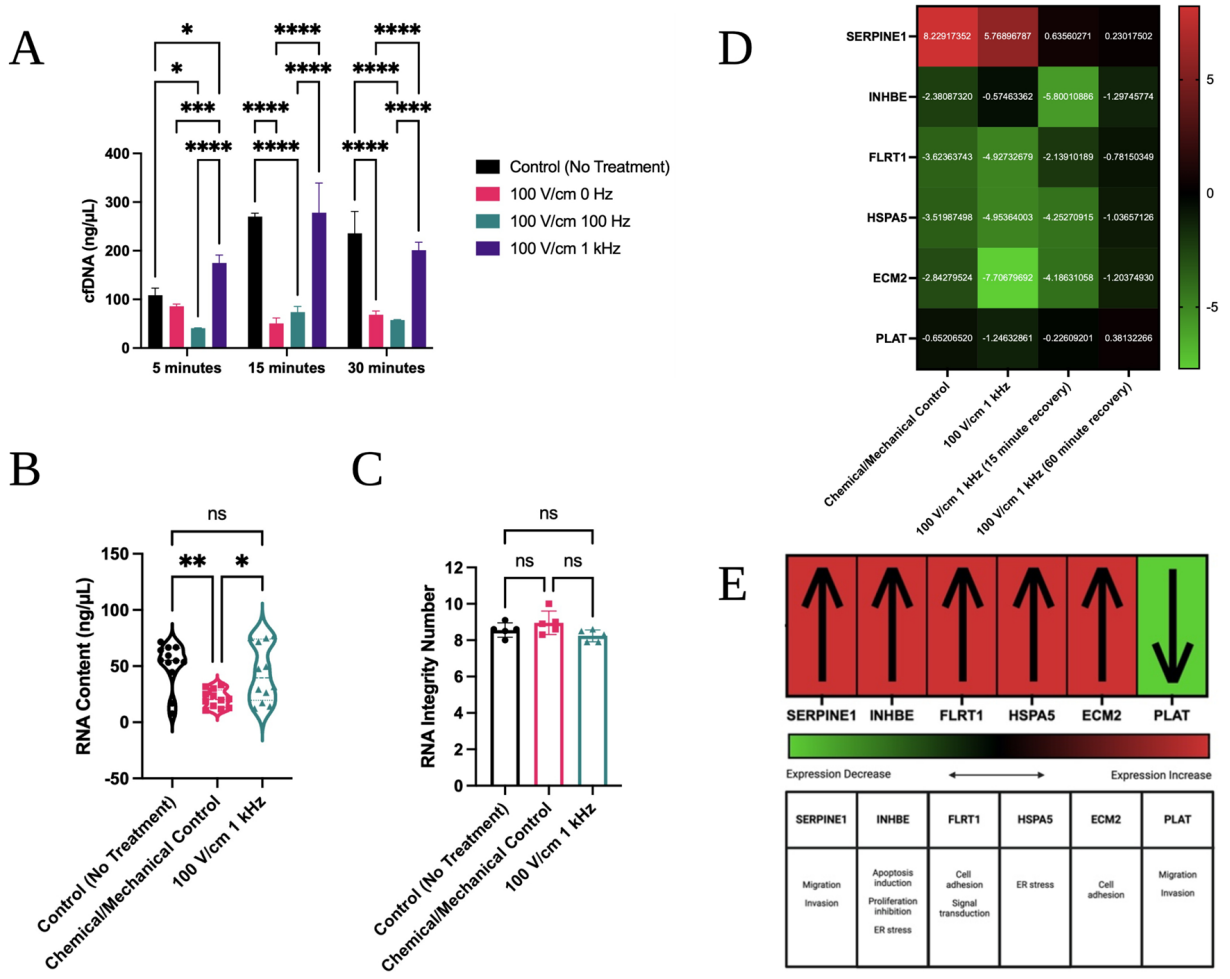
cfDNA in solution; extracted RNA quantity, characteristics and expression changes. (**A**) cfDNA in solution in ng/µL from whole tissue section after 5, 15, and 30 min of treatment with 100 V/cm at 0 Hz, 100 Hz and 1 kHz, or no treatment. Two-way ANOVA with Tukey post-hoc analysis and a 95% confidence interval was performed. (**B**) RNA content in ng/µL after RNA extraction from a starting population of 500,000 cells exposed to no treatment, a collagenase and mechanical agitation treatment, or the 100 V/cm 1 kHz treatment. One-way ANOVA with Tukey post-hoc analysis and a 95% confidence interval was performed (*p* = 0.0014). (**C**) RNA Integrity Number (RIN) for the extracted RNA samples. One-way ANOVA with Tukey post-hoc analysis and a 95% confidence interval was performed (*p* = 0.0941). (**D**) RT-qPCR expression profile changes for 6 indicators across treatments. ∆Cq was calculated for each treatment group by using a control expression baseline followingestablished guidelines^32^. Differential expression was then examinedwith a heatmap analysis and compared to (**E**) stress response signatures for the MDA-MB-231 cell line. *p < 0.05, **p < 0.01, ***p < 0.001, ****p < 0.0001.

cfDNA release was examined across 5-, 15-, and 30-min timepoints in an untreated control, as well as 100 V/cm electrical treatments at 0 Hz, 100 Hz and 1 kHz. It was found that the electrically treated conditions do not increase the concentration of cfDNA (Fig. 8A). From this preliminary data, it does not appear that cells leak their intracellular contents during processing, a necessary condition to translating this technology to SCS. Interestingly, an observed decrease in cfDNA content in the 0 Hz and 100 Hz conditions may indicate that cfDNA is disrupted at these electric field strengths and oscillation frequencies.

#### Effect of electric fields on RNA expression

The original, unadjusted RNA content was not significantly different in the 100 V/cm 1 kHz electrical treatment when compared to the control (Fig. 8B). However, the chemical/mechanical treatment had a slightly lower RNA content when compared to both other groups. All RIN values were 8 or above, consistent with intact RNA (Fig. 8C).

RT-qPCR results showed that a stress response was not observed in the chemical/mechanical or electrically treated cells, apart from the expression of migration and invasion genes SERPINE1 and PLAT, which increased and decreased, respectively (Fig. 8D,E). This is consistent with SERPINE1 serving as the principal inhibitor of PLAT, and furthermore an inhibitor of cellular migration^26^.

Notably, FLRT1 and ECM2, both cell adhesion markers, were downregulated in both the chemical/mechanical and electrically treated groups, but more so in the case of the electrical treatment with Cq_control_ - Cq_variable_ values of −3.62 versus −4.93 for FLRT1 and −2.84 versus −7.71 for ECM2, respectively (Fig. 8D). While this is not consistent with established trends of cellular stress in MDA-MB-231, it suggests a potential additional biological mechanism that could enhance tissue dissociation.

15- and 60-min recovery periods were investigated to determine if these expression trends could be reversed by putting the cells back into their preferred control conditions. Within 60 min, the cells can essentially return to their baseline levels, suggesting that the cells will be able to recover characteristic adhesion and migration properties.

## Discussion

This study represents the first characterization and analysis of electric field-facilitated dissociation of cells from tissue^28^. A complete physical mechanism by which the tissue dissociation phenomenon takes place may require additional investigation. However, pre-existing physical research on the application of electric fields to cellular suspensions can provide key insights into the process (Supplementary Information).

While electric fields and electrophoresis have been extensively studied in µ-scale biosensors and diagnostic devices^29^, the use of electrical forces is also commonly associated with electroporation and even cell death. The electric field is frequently used to form pores in cells in order to insert favorable genetic material, and has even been used to selectively kill cancer cells^30^.

However, electric fields can be a versatile tool in the laboratory. These results illustrate that voltages on the order of 2–20 V, with corresponding field strengths on the order of 10–100 V/cm and intermediate frequencies on the order of 10 Hz–1 kHz can be used to dissociate cells from tissues, including *ex vivo* tissues and *in vitro* cell aggregates.

Early results with non-oscillating voltages on shorter timescales helped to determine the contenders for optimum electric field conditions. While 100 V/cm had the highest sample purity and was seen to be the best condition for dissociating cells in the present study, cellular recovery was impacted by bubbling. This field strength was tested using oscillating voltage to optimize the process.

Flow cytometry results illustrated that 100 V/cm 1 kHz trials were the most effective at dissociating the tissue sections into cells within a 5-min timeframe. 95 ± 4% of entire 1 mm biopsy tissue sections were able to be dispersed into suspension with this condition.

Similarly, lower frequency oscillations could be as effective as the 1 kHz frequency in a longer timeframe, dissociating the entire tissue section into a cellular suspension. For a milder frequency dissociation, 500 Hz was observed to dissociate 91 ± 9% of cells from the tissue section within 30 min.

The electric field did not cause significant cellular fragmentation or cell death in MDA-MB-231 cells when compared to chemical and chemical/mechanical controls. In addition, mitosis and cell cycle progression were not significantly affected by 100 V/cm 1 kHz treatment. An increase in cfDNA was not observed during treatment nor were extracted RNA yield or RIN impacted. Gene expression profiling studies using RT-qPCR did not show significant stress response across the 6 investigated targets. Downregulation of cell adhesion markers such as ECM2 and FLRT1 was observed, suggesting a potential biological mechanism at play in the dissociation process. Control expression signatures could be recovered by placing cells in media within a temperature-controlled CO_2_ incubator. Human clinical GBM results show promise for translating the technique, however, it is likely that tissue-specific electrical parameter optimization will be necessary to adapt the technique to other tissue types and sizes.

Overall, the electrical tissue dissociation processes observed in this study suggest potential use as a platform for tunable, easy to use tissue dissociation preparations. The size scale is clinically applicable to small biopsy core sizes, while existing commercial products for disrupting tissues into single cells often use large volume sample preparation techniques that are poorly suited to the smaller biopsies often used in cancer diagnostics^31^. Hands-free tissue dissociation can be obtained by way of electric fields within a few minutes. This format may be amenable to high throughput scale-up of µm-cm scale tissue dissociation of multiple biopsy sections at once, potentially making it useful in Region of Interest (ROI) analyses from different tumors, tumor areas, or even different patients^31^.

## Conclusion

A rapid, low cost, miniaturized tissue dissociation methodology was demonstrated using applied electric fields to dissociate 1 mm biopsy tissue cores into cellular suspensions. This represents the first use of electric fields to dissociate tissues into cells^28^. The experimentally determined best electrical condition for tissue dissociation consisted of 100 V/cm at 1 kHz square wave frequency of oscillation. This condition was able to entirely dissociate 1 mm bovine liver tissue biopsy cores in 5 min without observable cell death, fragmentation, cell cycle disruption, or significant transcriptional stress response.

This technology is competitive with established chemical and mechanical protocols, which take 15 min at best to achieve similar dissociation results of the same tissue^6^. The electric field is a useful tissue dissociation tool that should be further explored from both a basic science and translational perspective.

## Supplementary Information

Supplementary Information.

## Data Availability

The data presented in the current study are available from the corresponding author upon reasonable request.
