# Supplementary material for: Electric-field facilitated rapid and efficient dissociation of tissues Into viable single cells
Source: Sci Rep. 2022 Jun 24;12:10728. doi: 10.1038/s41598-022-13068-6 (PMC9232619; doi:10.1038/s41598-022-13068-6)
Supplement: Supplementary file 1 — Supplementary Information. [file 41598_2022_13068_MOESM1_ESM.docx]

Electric-Field Facilitated Rapid and Efficient Dissociation of Tissues Into Viable Single Cells.

E. Celeste Welch^1^, Harry Yu^1^, Gilda Barabino^2^, Nikos Tapinos^3^, Anubhav Tripathi^1^

^1^Center for Biomedical Engineering, School of Engineering, Brown University, Providence, Rhode Island 02912.

^2^Franklin W. Olin College of Engineering, Needham, Massachusetts, 02492

^3^Department of Neurosurgery, Warren Alpert Medical School, Brown University, Providence, Rhode Island 02912.

Supplementary Information:

**Full Information on Physical Modeling of Electric Fields**

The AC/DC Module within COMSOL Multiphysics software was used for physical modeling of the dissociation phenomenon. 3D model geometries were designed using two parallel plate electrodes composed of aluminum, the 2 mm cavity filled with ultra-pure water, and a tissue cylinder model of the dimensions used in the study (diameter of 1 mm, height of 5 mm). Finite element analysis was performed by meshing the components using free triangular mesh with a minimum element size of 0.001 cm, although a grid independence study confirmed that the calculated solution was independent of the mesh size.

         The boundary conditions were set by defining the edge of the left electrode as the applied voltage while the second electrode was defined as the ground, as established in previous parallel plate electrical models^1^. The conductivity of the tissue cylinder within the cavity was set as 0.57 S/m, the known conductivity of healthy porcine liver as found in another study^2^. However, it is possible that temperature fluctuation could result in increases to the conductivity of the tissue, as also observed in this liver tissue conductivity study. A slightly higher value was used to accommodate any fluctuation. The aluminum electrode’s conductivity was 36.9x10^6 S/m and the conductivity of the ultra-pure water was 0.05 µS/cm as verified using a conductivity meter. An LCR meter was used to determine the dielectric constant of the LCMS grade H2O (78.4) by measuring the capacitance between the plates.

         The COMSOL results confirmed that the electric field strengths were as anticipated - for example, 10 V/cm for an applied voltage of 2 V, 100 V/cm for an applied voltage of 20 V, and so on. This provides insight into the optimum field strength for dissociation of tissue placed within that particular field. All voltages that were tested experimentally were also tested in COMSOL.

In addition to the tissue core at center model, dissociating tissue models were created within the cavity to simulate possible ways the electric field may change over time as the tissue core dissociates, and ensure field linearity across all trials. Layers were created (3-9) with different conductivities, ranging from solid tissue to ultra-pure water. Linear electric field lines were observed across all tested tissue models, favorably indicating that the tissue does not deflect the electric field in any significant manner, and that electric field hot spots are not created anywhere in the cavity (Figure 3).

Additionally, to assess whether these physical results would translate to the other electrical setups, the horizontally oriented setup was modeled in COMSOL and tested as well. Results were consistent across all electrical setups included.

**Dissociation Phenomena**

Negatively charged cells are moved by electrophoresis in a DC electric field^3^. In AC electric fields with high oscillation frequencies, dipole moments can be created and induce cellular orientation and shape-dependent phenomena such as electro-orientation and electro-rotation (Supplementary Equation S7)^3^.

Constant electric fields with low amplitudes have an established relationship with cellular movement and reorganization^4–7^. DC fields can induce redistribution of membrane channels and receptors and increase proliferation factor expression^7^. Additionally, decreases in adhesive forces have been observed under these conditions, as well as cytoskeletal reorganization and cellular migration^4–6^. However, the time scales and amplitudes tested in the presented work differ from existing studies.

In addition to limiting electrolysis, oscillating voltage limits sample heating and osmotic effects^3^. Higher frequencies of oscillation have been reported to cause deeper penetration of the electric field into cells, which could undesirably modulate nuclei and organelles. Moderate frequencies such as 1 kHz may have the ability to penetrate the tissue core but act mainly on surface proteins and adherence factors. However, pulsed oscillating voltage has been shown to result in protein expression changes^8,9^. This is potentially due to membrane voltage changes, or ion channel opening and closing, which can cause cellular expression changes^9,10^. Other studies have shown that oscillating electric fields can lower adhesion^8^.

The precise way in which electrokinetic processes can interact in complex, biologically active tissue systems is not yet apparent, although a clear relationship exists between electric field strength, oscillation frequency, and dissociation efficacy. Other research has characterized that electric fields and fluid shear forces share a common transduction mechanism in the production of large torques on transmembrane glycoproteins^11^. Furthermore, the induced bubble formation could serve as an additional mechanical dissociation force.

(Supplementary Equation S1)

$$Vc= \frac{3}{2}*r*Ec*cos\theta$$

Supplementary Equation S1 represents the Integrated Laplace Equation for a Spherical Cell in an Electric Field. Modified V_c_ can be assumed to be the membrane detachment voltage instead of the traditional definition of membrane breakdown voltage, as provided by Zimmermann and Vienken. r = cellular radius, E_c_ = critical electric field strength. θ = angle between location of cell and direction of electric field. All terms in the equation vary with individual cellular properties within the tissue core, making the problem highly complex.

**Physical Theory of Dissociation**

The physical theory of electrical tissue dissociation is based on extant physical research describing fragmentation phenomena. The basic fragmentation equation can be described, wherein a(x) is the rate of fragmentation of particles of a given size x that results when a particle of size y breaks up (Supplementary Equation S2)^23,24^. In this case, the particles can be considered the individual single cells, while the agglomerate can be considered the cellular aggregate or tissue.

$\frac{\partial c(x, t)}{\partial t}= -a\left( x \right)n\left( x,t \right)+ \int_{x}^{\infty} a(y)\bar{b}(x\left| y \right)c\left( y,t \right)dy$ (Supplementary Equation S2)

The particle mass distribution is represented by n(x,t), and the distribution of daughter particle masses is represented by $\bar{b}\left( x \right| y)$. The continuous-mass-loss rate can also be added to the equation, as explored by Edwards et al. Fragmentation has also been explored using fragmentation number (Supplementary Equation S3).

$F_{t}= \frac{\tau}{\sigma_{t}}$ (Supplementary Equation S3)

Where the fragmentation number is the ratio of applied shear stress (τ) to the cohesive strength of the tissue or cellular aggregate (σ_t_)^25-27^. The cohesive strength of the tissue or cellular aggregates can be estimated using an additional equation (Supplementary Equation S4)^26^. In this equation, $\bar{\varepsilon}$ is the average porosity, while E_af_ represents the energy required to break-up the cell-cell interaction, and D_c_ is the average cell size.

$\sigma_{t}= 1.1 \times\frac{1-\bar{\varepsilon}}{\bar{\varepsilon}}\frac{E_{tf}}{D_{c}^{3}}$ (Supplementary Equation S4)

**Cellular Recovery From Tissue**

(Supplementary Equation S5)

$$\frac{\left( V in {mm}^{3}*\left( \frac{cells}{{mm}^{3}} \right) \right)+(w in g*\left( \frac{cells}{g} \right))}{2}$$

Supplementary Equation S2 represents the basic equation for calculating the expected number of tissue cells within a bovine liver tissue based on weight and volume expected results previously established in the literature.

**Supplementary Figure S1 – Validation of Tissue Model Using Fully Dissociated Tissue Cores.**

**Part A) Representative Flow Cytometry Plot.**


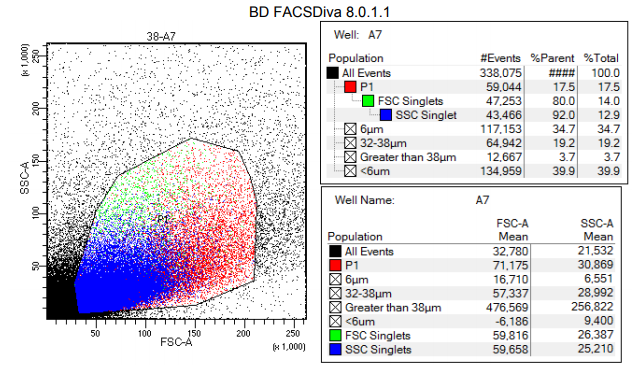


**Part B) For 1 Representative Tissue Section - Comparing Experimentally Determined Total Cell Count in Sample to Volume, Weight, and Hybrid Calculated Result.**

**
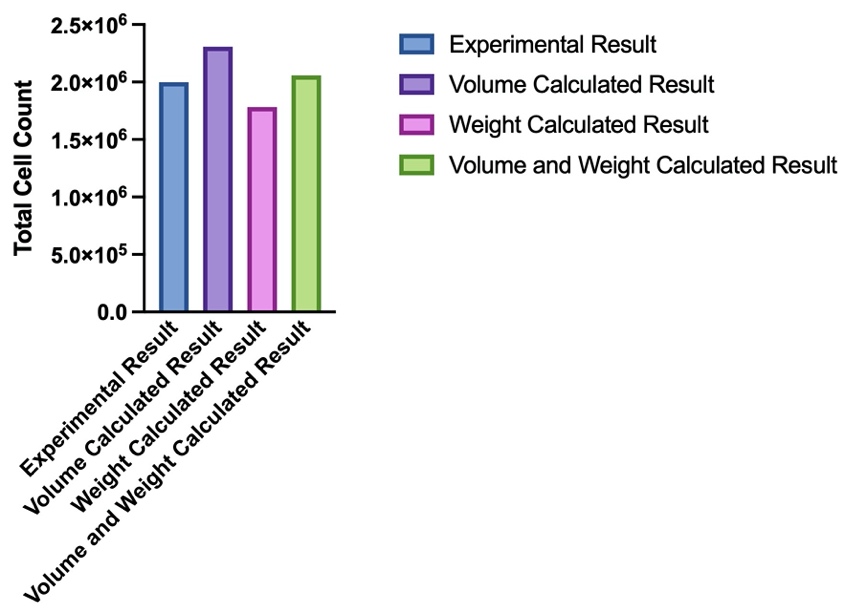
**

**Part C) Comparing Individual Volume and Weight Calculated Samples To Predicted Total Cell Count.**

**
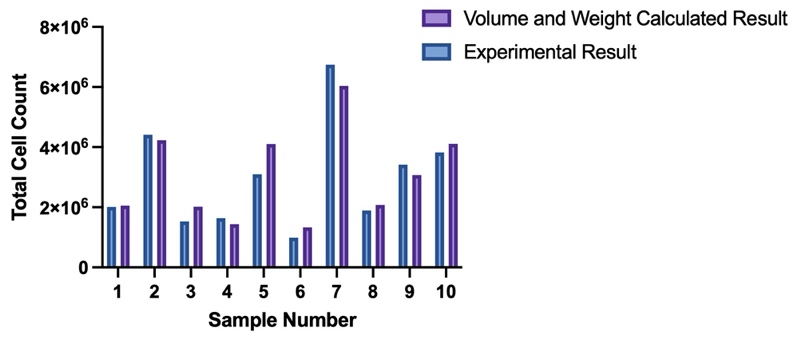
**

**Part D) Correlation Results Between The Calculated and Experimental Result.**

**
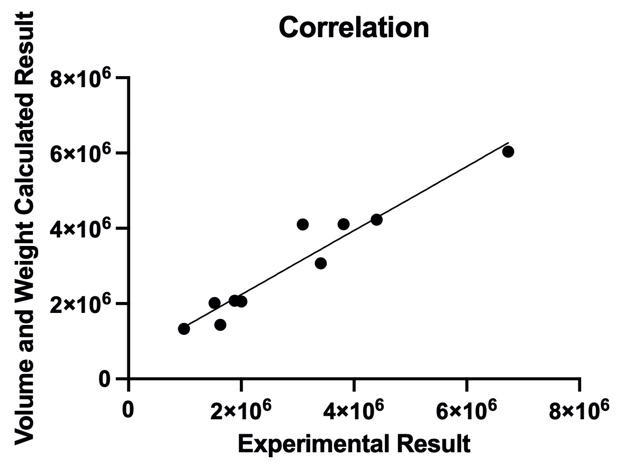
**

**Supplementary Figure S2 – Media Effect on Liquid Sample Recovery.**

**Part A) Sample Volume Loss To Bubbling Across Various DC Electric Field Strengths in H2O and Media Trials. Expressed in Percent Recovery of Sample Volume (%).**

**Part B) Sample Volume Loss To Bubbling Across Various Frequencies of Oscillation in H2O and Media Trials. Expressed in Percent Recovery of Sample Volume (%).**


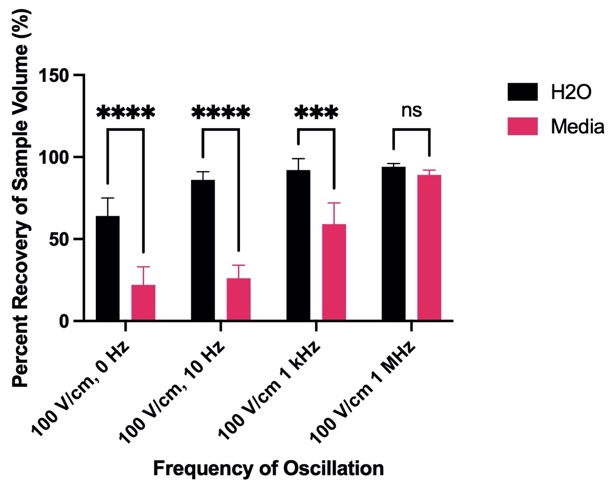


Two-way ANOVA with Tukey post-hoc analysis and a 95% confidence interval was performed for samples at the 5-minute time point. N ≥ 10, * p < 0.05, ** p < 0.01, ***p < 0.001, **** p < 0.0001.

**Supplementary Figure S3 – Media Effect on Cellular Viability During Electrical Treatment At The 5 Minute Timepoint.**

**
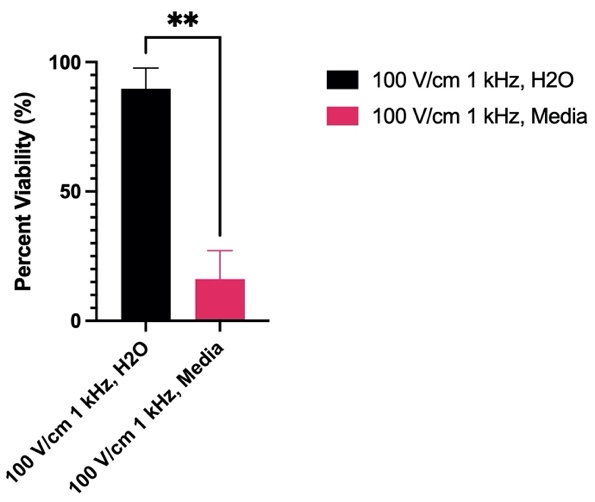
**

In addition to assessing liquid sample recovery, it was necessary to examine the effects of media with optimized electrical treatment on cellular viability. Unpaired t-Test with Welch’s correction. ** p < 0.01.

**Supplementary Figure S4 – Media Effect on Cellular Viability and Recovery Over Time Without Electrical Treatment.**

**
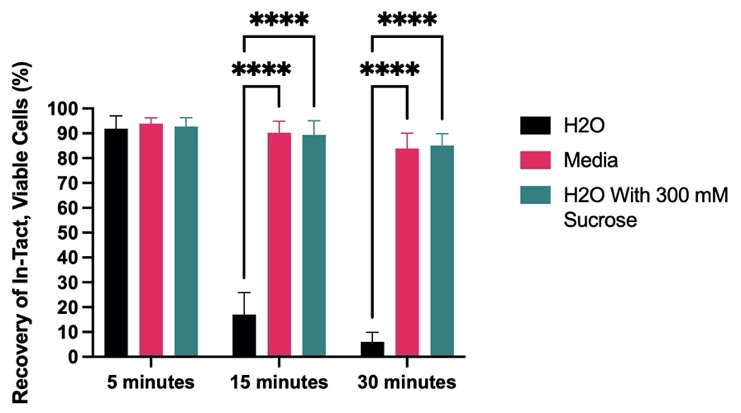
**

Media alone was assessed without any electrical treatment in order to determine the effect of media on viability decline. Two-way ANOVA with Tukey post-hoc analysis and a 95% confidence interval was performed for samples across all timepoints. N ≥ 10, * p < 0.05, ** p < 0.01, ***p < 0.001, **** p < 0.0001.

**Supplementary References**

1. Machado, M. C. *et al.* Rapid electrophoretic recovery of DNA from dried blood spots. *Electrophoresis* (2019) doi:10.1002/elps.201800363.

2. Zurbuchen, U. *et al.* Determination of the temperature-dependent electric conductivity of liver tissue ex vivo and in vivo: Importance for therapy planning for the radiofrequency ablation of liver tumours. *Int. J. Hyperth.* **26**, (2010).

3. Markx, G. H. The use of electric fields in tissue engineering. *Organogenesis* **4**, (2008).

4. Titushkin, I. & Cho, M. Regulation of Cell Cytoskeleton and Membrane Mechanics by Electric Field: Role of Linker Proteins. *Biophys. J.* **96**, (2009).

5. Brown, M. J. & Loew, L. M. Electric field-directed fibroblast locomotion involves cell surface molecular reorganization and is calcium independent. *J. Cell Biol.* **127**, (1994).

6. Onuma, E. K. & Hui, S. W. Electric field-directed cell shape changes, displacement, and cytoskeletal reorganization are calcium dependent. *J. Cell Biol.* **106**, (1988).

7. Fitzsimmons, R. J., Farley, J. R., Adey, W. R. & Baylink, D. J. Frequency dependence of increased cell proliferation, in vitro, in exposures to a low-amplitude, low-frequency electric field: Evidence for dependence on increased mitogen activity released into culture medium. *J. Cell. Physiol.* **139**, (1989).

8. Song, G., Qin, J., Yao, C. & Ju, Y. Effect of steep pulsed electric field on proliferation, viscoelasticity and adhesion of human hepatoma SMMC-7721 cells. *Anticancer Res.* **28**, 2245–2251 (2008).

9. Taghian, T., Narmoneva, D. A. & Kogan, A. B. Modulation of cell function by electric field: a high-resolution analysis. *J. R. Soc. Interface* **12**, (2015).

10. Sjaastad, M. D. & Nelson, W. J. Integrin-mediated calcium signaling and regulation of cell adhesion by intracellular calcium. *BioEssays* **19**, (1997).

11. Hart, F. X. & Palisano, J. R. The Application of Electric Fields in Biology and Medicine. in *Electric Field* (InTech, 2018). doi:10.5772/intechopen.71683.

12. Volovitz, I. *et al.* A non-aggressive, highly efficient, enzymatic method for dissociation of human brain-tumors and brain-tissues to viable single-cells. *BMC Neurosci.* **17**, (2016).

13. Welch, E. C., Yu, H. & Tripathi, A. Optimization of a Clinically Relevant Chemical-Mechanical Tissue Dissociation Workflow for Single-Cell Analysis. *Cell. Mol. Bioeng.* (2021) doi:10.1007/s12195-021-00667-y.

14. Waymouth, C. *Tissue Dissociation Guide*. (1993).

15. Lin, C.-H., Lee, D.-C., Chang, H.-C., Chiu, I.-M. & Hsu, C.-H. Single-Cell Enzyme-Free Dissociation of Neurospheres Using a Microfluidic Chip. *Anal. Chem.* **85**, (2013).

16. Wallman, L. *et al.* Biogrid—a microfluidic device for large-scale enzyme-free dissociation of stem cell aggregates. *Lab Chip* **11**, (2011).

17. Qiu, X. *et al.* Microfluidic filter device with nylon mesh membranes efficiently dissociates cell aggregates and digested tissue into single cells. *Lab Chip* **18**, (2018).

18. Qiu, X., De Jesus, J., Pennell, M., Troiani, M. & Haun, J. B. Microfluidic device for mechanical dissociation of cancer cell aggregates into single cells. *Lab Chip* **15**, (2015).

19. Qiu, X. *et al.* Microfluidic channel optimization to improve hydrodynamic dissociation of cell aggregates and tissue. *Sci. Rep.* **8**, (2018).

20. Qiu, X. *et al.* Microfluidic device for rapid digestion of tissues into cellular suspensions. *Lab Chip* **17**, (2017).

21. Miller, T. E., Mack, S. C. & Rich, J. N. *Mouse cell depletion from patient-derived xenograft brain tumors and isolation of glial cancer stem cells*. (2015).

22. Welch, E. C. & Tripathi, A. ELECTRICAL DISSOCIATION OF TISSUE SAMPLES INTO SINGLE CELLS AND/OR SMALLER GROUPS OF CELLS. (2021).

23. R. M. Ziff, “New Solutions To The Fragmentation Equation,” *J. Phys. A. Math. Gen.*, vol. 24, no. 12, p. 2821, 1991.

24. B. F. Edwards, M. Cai, and H. Han, “Rate equation and scaling for fragmentation with mass loss,” *Phys. Rev. A*, vol. 41, no. 10, pp. 5755–5757, May 1990, doi: 10.1103/PhysRevA.41.5755.

25. S. P. Rwei, I. Manas-Zloczower, and D. L. Feke, “Observation of carbon black agglomerate dispersion in simple shear flows,” *Polym. Eng. Sci.*, vol. 30, no. 12, pp. 701–706, Jun. 1990, doi: 10.1002/pen.760301202.

26. Y. Asanuma, F. Faizal, M. P. Khairunnisa, and I. W. Lenggoro, “Deagglomeration of spray-dried submicron particles by low-power aqueous sonication,” *Adv. Powder Technol.*, vol. 33, no. 4, p. 103543, Apr. 2022, doi: 10.1016/j.apt.2022.103543.

27. J. Bałdyga, Ł. Makowski, W. Orciuch, C. Sauter, and H. P. Schuchmann, “Deagglomeration processes in high-shear devices,” *Chem. Eng. Res. Des.*, vol. 86, no. 12, pp. 1369–1381, Dec. 2008, doi: 10.1016/j.cherd.2008.08.016.
